# A typical case of herpes zoster

**DOI:** 10.11604/pamj.2024.47.194.43003

**Published:** 2024-04-16

**Authors:** Souvik Sarkar, Sandeep Reddy

**Affiliations:** 1 Department of Respiratory Medicine, Datta Meghe Institute of Higher Education and Research, Wardha, Maharashtra, India

**Keywords:** Herpes zoster, varicella zoster, shingles

## Image in medicine

A 59-year-old male presented to the outpatient department with a painful rash on the lateral aspect of the thorax, fever with chills, and occasional cough for 1 week. He was a previously diagnosed case of a human immunodeficiency virus (HIV) infection and was taking irregular anti-retroviral therapy (ART). The patient was also a habitual alcoholic and had a past history of tuberculosis 2 years back. On examination, he was emaciated, afebrile to touch, had a pulse rate of 88 beats per minute, and a blood pressure of 90/60 mmHg. On examination of the rash, there was a dermatomal distribution along the lateral aspect of the right hemithorax. It had multiple erythematous papules, with some grouped vesicles and hemorrhagic pustules. The CD4 count of the patient was 114. The patient was initiated on oral acyclovir 800 mg five times daily, oral gabapentin 100 mg three times daily, and local application of lidocaine ointment. He was also restarted on regular anti-retroviral drugs, and gradually, the pain of the patient was relieved, and the rashes decreased, leaving a scar.

**Figure 1:**
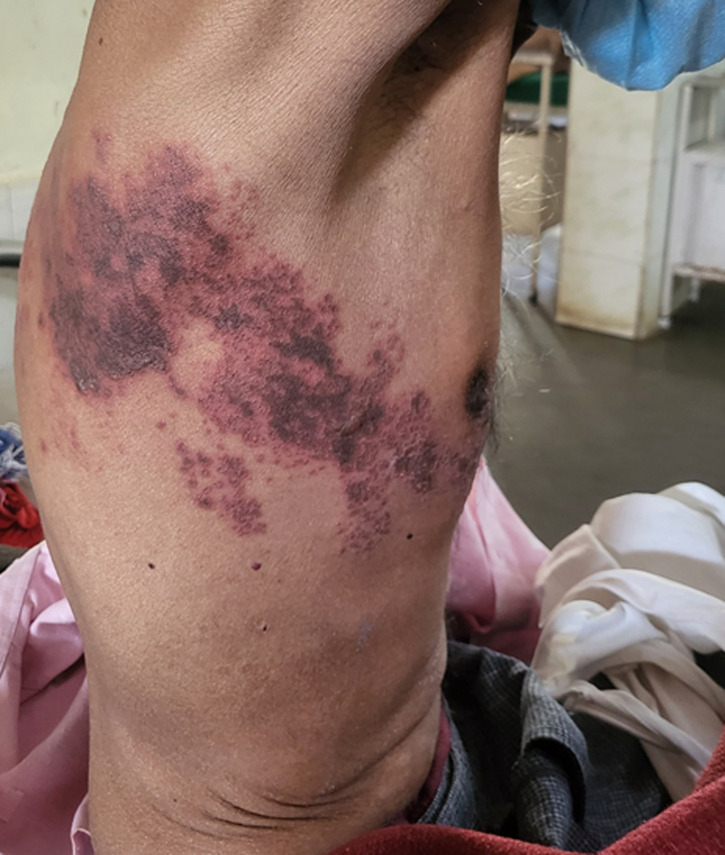
erythematous papules, with grouped vesicles and hemorrhagic pustules on the lateral aspect of the right hemithorax

